# Editorial: Novel Small-Molecule Agents in Overcoming Multidrug Resistance in Cancers

**DOI:** 10.3389/fchem.2022.921985

**Published:** 2022-05-04

**Authors:** Qingbin Cui, Cong Wang, Leli Zeng, Qian-Xiong Zhou, Ying-Fang Fan

**Affiliations:** ^1^ Department of Cell and Cancer Biology, University of Toledo College of Medicine and Life Sciences, Toledo, OH, United States; ^2^ State Key Laboratory of Esophageal Cancer Prevention and Treatment, School of Pharmaceutical Sciences, Zhengzhou University, Zhengzhou, China; ^3^ Guangdong Provincial Key Laboratory of Digestive Cancer Research, Scientific Research Center, The Seventh Affiliated Hospital, Sun Yat-sen University, Shenzhen, China; ^4^ Key Laboratory of Photochemical Conversion and Optoelectronic Materials, Technical Institute of Physics and Chemistry, Chinese Academy of Sciences, Beijing, China; ^5^ Department of First Hepatobiliary Surgery, Zhujiang Hospital, Southern Medical University, Guangzhou, China

**Keywords:** multidrug resistance, mechanism, reversal agents, medicinal chemistry, drug discovery

Multidrug resistance (MDR) is a term describing the phenomenon that cancer cells show resistant properties to various anticancer drugs of structurally and mechanistically different, accounting for approximate 90% of cancer treatment failures (Chaffer and Weinberg, 2011; Pluchino et al., 2012; Gao et al., 2020; Wang et al., 2021). It is worth noting that even the cutting-edge immunotherapy, is only 10%–20% effective, encountering resistance as well in certain patients (Sabbatino et al., 2018; Bai et al., 2020; Imbert et al., 2020; Bashash et al., 2022). Thus, MDR appears to be one of the major challenges in cancer treatment, which significantly undermines patients’ survival and quality of life (Zugazagoitia et al., 2016). The causes of MDR are usually multi-facet (Assaraf et al., 2019; Vasan et al., 2019), rendering it an even tougher challenge to tackle. Therefore, in the current Research Topic “Novel Small-Molecule Agents in Overcoming Multidrug Resistance in Cancers,” we attempted to collect the most-recent progress made in identifying new targets and agents in the treatment of drug-resistant cancers. In total, after being peer-reviewed, 20 manuscripts, composed with 13 research articles and seven review articles authored by 187 researchers worldwide, were successfully accepted for publication.

Natural products-derived drug discovery remains a practical and feasible strategy in the term of efficiently finding new hit/lead compounds that can undergo structural modification (Munos, 2009; Newman and Cragg, 2020). Guo et al. reviewed the latest progress of using eight classes of natural products in cancers. In their review, compounds of alkaloids, terpenoids, naturally occurring inorganic salt, phenylpropanoids, flavonoids, quinonoids, saponin and polysaccharides, their derivatives, as well as their research in cancer treatment, and the associated action modes were summarized and discussed. Specially, a separate review by Sun et al. from the same group above focused on matrine, a quinolizidine alkaloid isolated from *Sophora flavescentis*. Pharmacological effects including anticancer of matrine and its derivatives were summarized. In addition, critical structure-activity relationship (SAR) was also presented and discussed. Ji et al. conducted an interesting comparison of the flavones apigenin and genistein, two isomers that show similar yet different activities and mechanisms in prostate cancer. Importantly, genistein has been extensively studied in lab and in clinical trials for many diseases including cancers (Russo et al., 2016; Yu et al., 2021), and it has been used as nutritional supplement on market for decades. Genistein appears to be a more promising drug candidate and lead compound than its counterpart apigenin, since much more combinational regimens were developed and evaluated using genistein as a chemo-sensitizer. Deng et al. presented a systematic review of the recent progress of using natural diterpenoids in drug-resistant cancers cells overexpressing ATP-binding cassette (ABC) transporter. Jatrophanes, lathyranes, clerodanes, pimaranes, ingenanes, briaranes, segetane, jatropholane, pseudolaric acid, taxanes and euphoractine were thoroughly reviewed for their bioactivities in killing drug resistant cancer cells, and sensitizing certain chemotherapeutics.

Drug repurposing is another efficient strategy in identifying novel agents by defining new indications (Parvathaneni et al., 2019; Berdigaliyev and Aljofan, 2020; Dinić et al., 2020). Xu et al. summarized the anticancer activity of local anesthetic ropivacaine. Ropivacaine, at either clinic-relevant dose/concentrations or much higher concentrations, could suppress the proliferation, migration of certain drug-resistant cancer cells, including leukemia stem cells, triple-negative breast cancer MDA-MB-231 cells, and it sensitized 1) 5-fluorouracil (5-FU) in breast cancer MDA-MB-468 and SkBr cells, and 2) tumor necrosis factor α (TNFα) in human hepatoma HepG2 cells, human colon cancer HT-29 cells and human leukemic monocyte THP-1 cells. Chen et al. found that non-steroidal anti-inflammatory drug (NSAID) meclofenamic acid was a potential dual inhibitor of breast cancer resistance protein (BCRP) and multidrug resistance protein-7 (MRP-7), two important ABC transporters. Meclofenamic acid and its combination with gefitinib down-regulated the expression of both BCRP and MRP-7 in gefitinib-resistant non-small cell lung cancer (NSCLC) PC9-GR and H292-GR cell, mediated by FTO/m6A-demethylation/c-Myc axis. Meclofenamic acid could increase the intracellular concentration of gefitinib, leading to its re-sensitization.

Several compounds were found to show inhibitory effects toward drug resistant cancer cells, and synergistic effects were achieved when combined with certain chemotherapeutics. Ji et al. reported the recent progress of their novel phosphatidylinositide 3-kinase (PI3K) inhibitor XH30 in the treatment of temozolomide-resistant glioblastoma. XH30 was not only highly toxic toward temozolomide-resistant U251/TMZ and T98G cells *in vitro* and *in vivo*, but also was able to sensitize temozolomide in the U251/TMZ xenograft model, probably mediated by the negative regulation of transcription factor GLI1. In addition to its ability in suppressing brain metastasis (Ji et al., 2022), XH30 represents a promising drug candidate in combating drug resistance in glioblastoma. Liu et al. reported that the dual PI3K/mTOR inhibitor, DHW-221 could act as an inhibitor of P-gp (one of the most important ABC transporters which cause MDR) in resistant NSCLC A549/Taxol cells. DHW-221 was predicted to bind and inhibit P-gp, leading to down-regulated P-gp expression and enhanced cytotoxicity of taxol (a substrate of P-gp) in A549/Taxol cells. DHW-221 appeared to preferably target A549/Taxol cells over sensitive A549 cells, inducing cell death mediated by cell arrest, mitochondrial apoptosis pathway and mitogen-activated protein kinase (MAPK) pathway. More importantly, DHW-221 was able to suppress the migration and invasion of A549/Taxol cells, and it reduced tumor growth in A549/Taxol xenograft model, suggesting its great promise as a chemo-sensitizer and drug candidate. Anlotinib, a vascular endothelial growth factor receptor 2 (VEGFR2) inhibitor, is an approved anticancer drug in China for the treatment of locally advanced or metastatic NSCLC. Wang et al. further validated that it could act as a P-gp inhibitor. Anlotinib inhibited the efflux function of P-gp without altering its expression level and localization, leading to increased accumulation of chemotherapeutics that are P-gp substrates, thereby sensitizing doxorubicin, paclitaxel and vincristine in P-pg overexpressing osteosarcoma cells. In KHOSR2 xenograft model, the combination of anlotinib and docorubicin showed higher inhibitory effects as compared to mono-therapy, and no significant toxic effects were observed. Another drug ribociclib, a CDK4/6 inhibitor, was identified as a P-pg inhibitor by Zhang et al. Different with anlotinib, ribociclib could down-regulate the translation and transcription levels of P-gp, leading to increased concentration of colchicine and doxorubicin in P-gp overexpressing human epidermoid carcinoma KB-C2 cells. Both ribociclib and anlotinib could stimulate the ATPase of P-gp. Huang et al. reported the therapeutic effects of PI3K inhibitor LY294002 in Fms-like tyrosine kinase 3-internal tandem duplication (FLT3-ITD) mutant acute myeloid leukemia (AML) cells that show sorafenib-resistant property. LY294002 inhibited the glycolysis of sorafenib-resistant cells which rely heavily on glycolysis for ATP production, leading to cell apoptosis. Liu et al. designed and evaluated a series of dual c-Met/VEGFR-2 inhibitors based on the core structure triazolopyrazine. Their study yielded a highly potent compound 17l that can suppress the enzymatic activities of c-Met and VEGFR-2, and inhibit the proliferation of A549 cells. Cao et al. found that SAHA, a histone deacetylase inhibitor, when combined with gefitinib or osimertinib, showed synergistic effects in the corresponding resistant NSCLC PC-9/AB2 and H1975OR cells *in vitro* and in animal models (SAHA plus gefitinib), which was mediated by increased enhancer of zeste homolog 2 (EZH2) and decreased autophagy. Heat shock protein 90 (HSP90) inhibitor, NVP-AUY922 was terminated in phase II clinical trials, despite its favorable results in overcoming drug resistance NSCLC cells. He et al. obtained the co-crystallization of NVP-AUY922 with HSP90, which showed detailed binding information that further directed the further drug design. Wu et al. designed and synthesized a novel murine double minute 2 (MDM2) inhibitor, XR-2 that was able to disturb the MDM2-p53 interaction, induce cell cycle arrest and apoptosis of wild-type p53 castration-resistant prostate cancer (CRPC) cell lines. In the enzalutamide-resistant CRPC xenograft model, XR-2 showed a strong synergistic effect with enzalutamide, resulting in a nearly complete inhibition of tumor growth while without showing any toxic effects.

In addition, several potential druggable targets causing drug resistance were also highlighted. Fu et al. found that the overexpression of P-gp can cause resistance of approved drug CDK 4/6 inhibitor palbociclib, whereas the inhibition of the efflux of P-gp by verapamil could restore its sensitivity. Similar phenomenon was also observed by Dong et al. on ARS-1620, a KRAS-G12C inhibitor that is now under clinical trials. Furthermore, Li et al. reported the involvement of high mobility group box protein 1 (HMGB1) in doxorubicin resistance, and its inhibitor ethyl pyruvate that can significantly enhance the sensitivity of doxorubicin in HMGB1 overexpressing hepatocellular carcinoma BEL7402 and SMMC7721 cells. Yu et al. summarized the druggability of mitochondrial DNA-directed RNA polymerase (POLRMT) and its inhibitors in overcoming drug resistance. Liu et al. systematically reviewed circular RNAs (circRNAs) and their roles in inducing drug resistance.

In conclusion, the identified novel small-molecule agents can serve as drug candidates and/or leading compounds that can be further structurally modified. Furthermore, rational designed combinations using these agents should be evaluated in the future to combat drug resistance.

## References

[B1] Assaraf Y. G. Brozovic A. Gonçalves A. C. Jurkovicova D. Linē A. Machuqueiro M. (2019). The Multi-Factorial Nature of Clinical Multidrug Resistance in Cancer. Drug Resist. Updat. 46, 100645. Available at: http://www.ncbi.nlm.nih.gov/entrez/query.fcgi?cmd=Retrieve&db=pubmed&dopt=Abstract&list_uids=31585396&query_hl=1 . 10.1016/j.drup.2019.100645 31585396

[B2] Bai R. Chen N. Li L. Du N. Bai L. Lv Z. (2020). Mechanisms of Cancer Resistance to Immunotherapy. Front. Oncol. 10, 1290. Available at: http://www.ncbi.nlm.nih.gov/entrez/query.fcgi?cmd=Retrieve&db=pubmed&dopt=Abstract&list_uids=32850400&query_hl=1 . 10.3389/fonc.2020.01290 32850400 PMC7425302

[B3] Bashash D. Zandi Z. Kashani B. Pourbagheri‐Sigaroodi A. Salari S. Ghaffari S. H. (2022). Resistance to Immunotherapy in Human Malignancies: Mechanisms, Research Progresses, Challenges, and Opportunities. J. Cell. Physiology 237 (1), 346–372. Available at: http://www.ncbi.nlm.nih.gov/entrez/query.fcgi?cmd=Retrieve&db=pubmed&dopt=Abstract&list_uids=34498289&query_hl=1 . 10.1002/jcp.30575 34498289

[B4] Berdigaliyev N. Aljofan M. (2020). An Overview of Drug Discovery and Development. Future Med. Chem. 12 (10), 939–947. Available at: http://www.ncbi.nlm.nih.gov/entrez/query.fcgi?cmd=Retrieve&db=pubmed&dopt=Abstract&list_uids=32270704&query_hl=1 . 10.4155/fmc-2019-0307 32270704

[B5] Chaffer C. L. Weinberg R. A. (2011). A Perspective on Cancer Cell Metastasis. Science 331 (6024), 1559–1564. Available at: http://www.ncbi.nlm.nih.gov/entrez/query.fcgi?cmd=Retrieve&db=pubmed&dopt=Abstract&list_uids=21436443&query_hl=1 . 10.1126/science.1203543 21436443

[B6] Dinić J. Efferth T. García-Sosa A. T. Grahovac J. Padrón J. M. Pajeva I. (2020). Repurposing Old Drugs to Fight Multidrug Resistant Cancers. Drug Resist. Updat. 52, 100713. Available at: http://www.ncbi.nlm.nih.gov/entrez/query.fcgi?cmd=Retrieve&db=pubmed&dopt=Abstract&list_uids=32615525&query_hl=1 . 10.1016/j.drup.2020.100713 32615525

[B7] Gao H.-L. Gupta P. Cui Q. Ashar Y. V. Wu Z.-X. Zeng L. (2020). Sapitinib Reverses Anticancer Drug Resistance in Colon Cancer Cells Overexpressing the ABCB1 Transporter. Front. Oncol. 10, 574861. Available at: http://www.ncbi.nlm.nih.gov/entrez/query.fcgi?cmd=Retrieve&db=pubmed&dopt=Abstract&list_uids=33163405&query_hl=1 . 10.3389/fonc.2020.574861 33163405 PMC7581728

[B8] Imbert C. Montfort A. Fraisse M. Marcheteau E. Gilhodes J. Martin E. (2020). Resistance of Melanoma to Immune Checkpoint Inhibitors is Overcome by Targeting the Sphingosine Kinase-1. Nat. Commun. 11 (1), 437. Available at: http://www.ncbi.nlm.nih.gov/entrez/query.fcgi?cmd=Retrieve&db=pubmed&dopt=Abstract&list_uids=31974367&query_hl=1 . 10.1038/s41467-019-14218-7 31974367 PMC6978345

[B9] Ji M. Wang D. Lin S. Wang C. Li L. Zhang Z. (2022). A Novel PI3K Inhibitor XH30 Suppresses Orthotopic Glioblastoma and Brain Metastasis in Mice Models. Acta Pharm. Sin. B 12 (2), 774–786. Available at: http://www.ncbi.nlm.nih.gov/entrez/query.fcgi?cmd=Retrieve&db=pubmed&dopt=Abstract&list_uids=35256946&query_hl=1 . 10.1016/j.apsb.2021.05.019 35256946 PMC8897175

[B10] Munos B. (2009). Lessons from 60 Years of Pharmaceutical Innovation. Nat. Rev. Drug Discov. 8 (12), 959–968. Available at: http://www.ncbi.nlm.nih.gov/entrez/query.fcgi?cmd=Retrieve&db=pubmed&dopt=Abstract&list_uids=19949401&query_hl=1 . 10.1038/nrd2961 19949401

[B11] Newman D. J. Cragg G. M. (2020). Natural Products as Sources of New Drugs Over the Nearly Four Decades from 01/1981 to 09/2019. J. Nat. Prod. 83 (3), 770–803. Available at: http://www.ncbi.nlm.nih.gov/entrez/query.fcgi?cmd=Retrieve&db=pubmed&dopt=Abstract&list_uids=32162523&query_hl=1 . 10.1021/acs.jnatprod.9b01285 32162523

[B12] Parvathaneni V. Kulkarni N. S. Muth A. Gupta V. (2019). Drug Repurposing: A Promising Tool to Accelerate the Drug Discovery Process. Drug Discov. Today 24 (10), 2076–2085. Available at: http://www.ncbi.nlm.nih.gov/entrez/query.fcgi?cmd=Retrieve&db=pubmed&dopt=Abstract&list_uids=31238113&query_hl=1 . 10.1016/j.drudis.2019.06.014 31238113 PMC11920972

[B13] Pluchino K. M. Hall M. D. Goldsborough A. S. Callaghan R. Gottesman M. M. (2012). Collateral Sensitivity as a Strategy against Cancer Multidrug Resistance. Drug Resist. Updat. 15 (1-2), 98–105. http://www.ncbi.nlm.nih.gov/entrez/query.fcgi?cmd=Retrieve&db=pubmed&dopt=Abstract&list_uids=22483810&query_hl=1. 10.1016/j.drup.2012.03.002 22483810 PMC3348266

[B14] Russo M. Russo G. L. Daglia M. Kasi P. D. Ravi S. Nabavi S. F. (2016). Understanding Genistein in Cancer: The "Good" and the "Bad" Effects: A Review. Food Chem. 196, 589–600. Available at: http://www.ncbi.nlm.nih.gov/entrez/query.fcgi?cmd=Retrieve&db=pubmed&dopt=Abstract&list_uids=26593532&query_hl=1 . 10.1016/j.foodchem.2015.09.085 26593532

[B15] Sabbatino F. Marra A. Liguori L. Scognamiglio G. Fusciello C. Botti G. (2018). Resistance to Anti-PD-1-Based Immunotherapy in Basal Cell Carcinoma: a Case Report and Review of the Literature. J. Immunother. Cancer 6 (1), 126. Available at: http://www.ncbi.nlm.nih.gov/entrez/query.fcgi?cmd=Retrieve&db=pubmed&dopt=Abstract&list_uids=30458852&query_hl=1 . 10.1186/s40425-018-0439-2 30458852 PMC6247622

[B16] Vasan N. Baselga J. Hyman D. M. (2019). A View on Drug Resistance in Cancer. Nature 575 (7782), 299–309. Available at: http://www.ncbi.nlm.nih.gov/entrez/query.fcgi?cmd=Retrieve&db=pubmed&dopt=Abstract&list_uids=31723286&query_hl=1 . 10.1038/s41586-019-1730-1 31723286 PMC8008476

[B17] Wang J.-Q. Yang Y. Cai C.-Y. Teng Q.-X. Cui Q. Lin J. (2021). Multidrug Resistance Proteins (MRPs): Structure, Function and the Overcoming of Cancer Multidrug Resistance. Drug Resist. Updat. 54, 100743. Available at: http://www.ncbi.nlm.nih.gov/entrez/query.fcgi?cmd=Retrieve&db=pubmed&dopt=Abstract&list_uids=33513557&query_hl=1 . 10.1016/j.drup.2021.100743 33513557

[B18] Yu T. Huang D. Wu H. Chen H. Chen S. Cui Q. (2021). Navigating Calcium and Reactive Oxygen Species by Natural Flavones for the Treatment of Heart Failure. Front. Pharmacol. 12, 718496. Available at: http://www.ncbi.nlm.nih.gov/entrez/query.fcgi?cmd=Retrieve&db=pubmed&dopt=Abstract&list_uids=34858167&query_hl=1 . 10.3389/fphar.2021.718496 34858167 PMC8630744

[B19] Zugazagoitia J. Guedes C. Ponce S. Ferrer I. Molina-Pinelo S. Paz-Ares L. (2016). Current Challenges in Cancer Treatment. Clin. Ther. 38 (7), 1551–1566. Available at: http://www.ncbi.nlm.nih.gov/entrez/query.fcgi?cmd=Retrieve&db=pubmed&dopt=Abstract&list_uids=27158009&query_hl=1 . 10.1016/j.clinthera.2016.03.026 27158009

